# Small Cell Oesophageal Carcinoma: A Retrospective Case Series From a UK Tertiary Centre and a Review of the Literature

**DOI:** 10.7759/cureus.49435

**Published:** 2023-11-26

**Authors:** Sari Zemerly, Krija Thurairajasingam, Bassam Deeb, Michael Tilby

**Affiliations:** 1 Oncology, University Hospitals Coventry and Warwickshire, Coventry, GBR; 2 Internal Medicine, Imperial College Trust, London, GBR; 3 Faculty of Medical Sciences, Lebanese University, Beirut, LBN

**Keywords:** radiotherapy (rt), platinum based chemotherapy, ec- esophageal cancer, oesophageal cancer, neuroendocrine carcinoma of esophagus, extrapulmonary neuroendocrine carcinoma, small-cell lung carcinoma, extra-pulmonary small cell carcinoma

## Abstract

Background

Small cell oesophageal carcinoma (SCEC), a rare neuroendocrine malignancy, presents various challenges in diagnosis and treatment. The condition is characterised by rapid dissemination, a marked responsiveness to chemotherapy, and a guarded prognosis. While the European Neuroendocrine Tumour Society has recommended platinum-based chemotherapy, ongoing debates on optimal strategies and the lack of clear guidelines underscore the need for further comprehensive research efforts.

Methods

This study retrospectively analysed 12 cases of localised pure SCEC treated at a UK tertiary care centre between 2006 and 2020. We systematically analysed and categorised the cases based on stage, performance status, and patient age. This comprehensive approach enabled a nuanced examination of overall survival (OS), thereby providing valuable insights into the differences between outcomes.

Results

The study revealed a median OS of 12.01 months for treated non-metastatic cases, highlighting the challenges of SCEC management. Conversely, treated metastatic cases exhibited a mean survival of 9.15 months, which contrasts starkly with the 2.55 months demonstrated by those receiving best supportive care (BSC). These figures underscore the urgency for refined strategies in handling advanced localised disease and the need to continue research endeavours to devise methods to enhance the precision and optimise outcomes beyond the presented data.

Conclusion

Based on our findings, the combination of chemoradiotherapy and surgery to manage SCEC holds promise; however, further research is needed to optimise the management approach. The lack of clear guidelines underscores the imperative for personalised treatment approaches.

## Introduction

Small cell cancer is an aggressive, high-grade neuroendocrine malignancy classically characterised by rapid dissemination and responsiveness to chemotherapy treatment [1]. It is primarily seen in the lungs but sporadically affects the ovaries, prostate, and gastrointestinal organs, including the oesophagus [2-3]. Histologically, small cell carcinoma manifests as small-sized, round cells exhibiting a thin cytoplasm and a finely granular nucleus frequently devoid of distinct nucleoli. Cellular necrosis and apoptosis are common, and the cells often exhibit an elevated mitotic rate [4-5]. Florence McKeown initially described two distinct cases of “oat-cell carcinoma” of the oesophagus on autopsy in 1952. This nomenclature has since become synonymous with the contemporary designation of "small cell carcinoma", emphasising the morphological resemblance to the appearance of oats under a light microscope [6]. Primary extrapulmonary small cell carcinoma notably shares histological traits with bronchogenic small cell disease and demonstrates a marked predilection to metastasis. Interestingly, secondary metastatic deposits have consistently been found to mirror the histological characteristics of the primary small cell malignancy. The true nature and histogenesis of small cell carcinoma remain a subject of controversy, with some reports suggesting a potential origin rooted in APUD cells, emphasising the need for further exploration and a comprehensive understanding of the fundamental mechanisms steering the progression of the malignant process [7-8].

Small cell oesophageal carcinoma (SCEC) is generally classified into two subtypes: "pure SCEC" and "combined SCEC". The latter is characterised by the histological presence of small cell oesophageal disease alongside additional cellular components such as adenocarcinoma, squamous cell carcinoma, or large cell carcinoma [4,9]. SCEC is a rare subtype, constituting 0.4-2.8% of all oesophageal malignancies, with a predilection for the middle and lower segments of the oesophagus [5,7,10]. It mainly affects elderly individuals and has a male-to-female ratio of 3:2. Its incidence is considerably higher in Far East countries such as Japan, Korea, and China [9]. The key risk factors include heavy smoking and alcohol consumption, as well as diets with high salt content. The latter may explain the increased prevalence of the disease in the Far East [8,10-11]. The clinical presentation generally resembles that of squamous cell carcinoma of the oesophagus. The primary symptoms include progressive dysphagia, accompanied by secondary manifestations such as odynophagia, anorexia, and weight loss. Furthermore, chest pain and vomiting may occur less frequently [5,10]. Approximately half of small cell oesophageal cancers are metastatic at the time of diagnosis, underscoring the critical role of staging in informing optimal treatment decisions for the effective management of the condition [10,12]. The adopted tumour staging strategies are based on those employed in the staging of small cell carcinoma of the lung [13].

Gastroscopy is commonly utilised for preoperative histological diagnosis [10,13]. The accuracy of this minimally invasive procedure is often compromised by the relatively small tissue volume obtained and the incidental coexistence of other tissue components, such as adenocarcinoma, squamous cell carcinoma, or non-small cell oesophageal carcinoma, within the same biopsy sample [10]. The comprehensive staging of the disease is executed using positron emission tomography (PET) and CT imaging of the thorax, abdomen and pelvis [2,11]. SCEC is associated with a poor prognosis and management approaches for this malignancy are frequently derived from practices employed in the treatment of small cell carcinoma of the lung due to several shared clinicopathological features [2-5,14]. For patients diagnosed with primary small cell oesophageal cancer with no evidence of distant metastasis, the spectrum of management options encompasses neoadjuvant chemotherapy, radiotherapy, and surgical resection [2-3,5,13-14]. The sequential integration of multiple treatment modalities has shown the potential to attain effective local control while mitigating the morbidity associated with an extensive surgical approach [13,15].

Surgery and adjuvant chemotherapy are efficacious at curtailing the dissemination of localised or locoregional disease [2,15-17]. Radiotherapy also plays a pivotal role and is often utilised to consolidate the primary site of the malignancy, either in a sequential manner following systemic treatment or concurrently with chemotherapy [2]. The incorporation of prophylactic cranial irradiation (PCI) is contentious and requires meticulous consideration and individualised assessment based on the unique health conditions of each patient [13]. The optimal choice between surgery and radiotherapy for the treatment of localised small cell oesophageal cancer remains a matter of debate [3,18]. Notably, exclusive dependence on radical oesophagectomy or radiotherapy appears insufficient. This emphasises the need for their discerning integration with adjuvant or neoadjuvant chemotherapy for a more comprehensive therapeutic impact [13].

The European Neuroendocrine Tumour Society proposes platinum-based chemotherapy such as cisplatin and carboplatin as a first-line systemic treatment for localised disease. Alternatively, a regimen involving a platinum compound with etoposide chemotherapy is primarily recommended in advanced or inoperable cases in patients with good performance status [2-3]. If subsequent chemotherapy is required on progression, as with small cell lung carcinoma, rechallenge with platinum-based chemotherapy is employed. Some studies have explored the administration of irinotecan or oxaliplatin-based formulations with cisplatin as a potential second-line treatment in progressive disease [3,14,19]. In cases of advanced-stage or metastatic illness, the administration of palliative chemotherapy has the potential to increase overall survival (OS) [3]. Moreover, radiotherapy can be used for local control in particular patients presenting with a relatively good performance status [13-14,18].

## Materials and methods

This retrospective series involves 12 distinctive cases of non-metastatic SCEC. The patients included in the study were British nationals and received their anti-cancer treatment at a prominent UK tertiary care centre between 2006 and 2020. The cases are systematically presented in accordance with their identified stage at diagnosis, performance status, and age. Essential information pertaining to the date of histological diagnosis, initiation of chemotherapy, and post-treatment imaging was methodically recorded. This systematic documentation served as the foundation for the subsequent computation of both overall and progression-free survival (PFS) rates, enabling thorough statistical examination and comprehensive evaluation of the therapeutic interventions employed. OS was calculated from the point of histological diagnosis to the recorded date of death, while PFS was determined by identifying the onset of clinical or radiological deterioration.

A detailed study of the chemotherapy regimens administered, including the total number of administered cycles and associated side effects, provided a holistic perspective of the treatment trajectory for each featured patient. Corresponding treatment responses were meticulously assessed through imaging, which offered valuable insights into disease progression or response to the delivered treatment. Subsequent cycles of chemotherapy, adjuvant radiotherapy, and supportive management were additionally noted, delineating the complexities of the management plan devised for each individual patient. Six instances featuring histological evidence of combined SCEC were excluded from this case series. Furthermore, four patients presenting with metastatic small cell cancer who received first-line chemotherapy were omitted from the case series as this study specifically focused on localised (or limited-stage) pure small cell carcinoma of the oesophagus. Nevertheless, the cases of treated metastatic disease are still incorporated into Table 1 to facilitate a comparative analysis of OS with advanced localised disease.

**Table 1:** Management Outcomes in Patients With Small Cell Oesophageal Carcinoma PS: performance status; Syn: synaptophysin status; CgA: chromogranin A status; CTx: chemotherapy; CB: carboplatin; EC: etoposide and cisplatin; EP: etoposide and carboplatin; CAV: cyclophosphamide, doxorubicin, and vincristine; PR: partial response; PD: disease progression; Rx: treatment; RT: radiotherapy; PCI: prophylactic cranial irradiation; PFS: progression-free survival (in months); OS: overall survival

	PS	TNM	Ki-67 %	Syn	CgA	1° CTx		1° Response	2° CTx		2° Response	Other Rx	PFS (Months)	OS (Months)
1	0	T2N1M0	N/A	Positive	N/A	EP	6 cycles	PR	None			Oesophagectomy	N/A	12.68 +
2	2	T2N1M0	N/A	N/A	Positive	EP	4 cycles	PD	CAV	1 cycle	PD	None	14.76	16.9
3	1	T3N0M0	>90%	Positive	Positive	EC	6 cycles	PR	None			RT (55Gy/20)	N/A	27.37 +
4	1	T3N0M0	N/A	Negative	Negative	EP	6 cycles	PR	EP	6 cycles	PR	3° Carboplatin (6 cycles)	12.2	38.26 +
5	0	T3N1M0	100	Positive	N/A	EC	6 cycles	PR	CAV	4 cycles	PD	RT (50.4Gy/28) and PCI	8.75	16.54
6	0	T3N1M0	N/A	Positive	Negative	EP	1 cycle	PD	CAV	1 cycle	PD	Stent	0.59	5.56
7	1	T3N1M0	90	Positive	Negative	EC	5 cycles	PR	None			None	N/A	4.18
8	0	T3N2M0	90	Positive	N/A	EP	3 cycles	N/A	None			RT (50Gy/28) and PCI	N/A	11.34
M1	1	T3N2M1	N/A	N/A	Positive	EP	5 cycles	PD	None			None	2.66	5.85
9	1	T4N0M0	N/A	Positive	N/A	Carboplatin	1 cycle	PD	None			RT (20Gy/5) and NJ Tube	1.15	5.16
10	1	T4N1M0	N/A	Positive	Negative	EP	6 cycles	PR	None			None	6.08	10.75
11	2	T4N1M0	N/A	Positive	Positive	Carboplatin	2 cycles	PD	None			Stent	1.84	4.18
12	0	T4N2M0	N/A	Positive	Positive	EP	5 cycles	PR	None			None	8.19	21.86
M2	1	T4N3M1	N/A	Positive	Positive	EP	6 cycles	PR	None			None	4.14	5.69
M3	1	T4N3M1	N/A	Positive	Negative	EP	6 cycles	PD	CAV	3 cycles	PD	None	4.37	10.55
M4	1	T4N3M1	N/A	Positive	N/A	EP	6 cycles	PR	None			None	13.58	14.5

Finally, a comparison was drawn by juxtaposing the selected cohort with a broader group of 16 other patients afflicted with metastatic SCEC who received best supportive care (BSC) as an integral part of their cancer management. Such a comprehensive approach enabled a more holistic interpretation of the suggested findings, contributing to the overall depth and applicability of our research insights.

## Results

Case 1

An individual was diagnosed with T2N1 small cell oesophageal cancer and started on six cycles of carboplatin and etoposide. They suffered from neutropenia, nausea, and vomiting as a side effect of chemotherapy. Repeat imaging revealed a partial response to the treatment. They subsequently underwent a radical oesophagectomy and suffered from no documented disease progression later. The patient remained alive at the conclusion of this study, with a survival period of at least 12.68 months from the time of initial diagnosis.

Case 2

An elderly patient was diagnosed with T2N1 small cell oesophageal carcinoma. They received four cycles of carboplatin and etoposide over the ensuing year without any significant reported treatment-related side effects. Post-treatment imaging revealed disease progression. This prompted the introduction of cyclophosphamide, doxorubicin, and vincristine (CAV) as a second-line chemotherapy option. Further disease progression was observed within two months, culminating in the patient's death 16.90 months from the initial diagnosis.

Case 3

A patient was diagnosed with T3N0 disease and treatment with carboplatin and etoposide was initiated. They received six cycles with no major side effects. Repeat imaging revealed a partial response with no evidence of further disease progression. The patient subsequently received a total of 55 Gy of radical radiation therapy to the oesophagus over 20 individual sessions to consolidate the response to the neoadjuvant chemotherapy administered. The patient was alive at the conclusion of this study, revealing an OS of at least 27.37 months from the time of initial diagnosis.

Case 4

An elderly individual was diagnosed with T3N0 small cell oesophageal cancer. They Initially underwent five cycles of carboplatin and etoposide, but the treatment was complicated by cytopenia and fatigue. Subsequent imaging demonstrated a partial response. The patient's PFS was noted at 12.20 months, leading to a rechallenge with platinum-based chemotherapy. Over four months, an additional six cycles led to a further partial response. Unfortunately, the disease progressed thereafter, necessitating third-line treatment of six cycles of carboplatin alone. Follow-up imaging displayed evidence of stable disease with no further progression. The patient remained alive at the conclusion of this study, showing a survival period of at least 38.26 months from the initial histological diagnosis.

Case 5

A middle-aged patient was diagnosed with T3N1M0 small cell oesophageal cancer. They underwent six cycles of treatment with cisplatin and etoposide over the course of three months with minimal documented side effects. CT imaging demonstrated a partial response to the chemotherapy. They subsequently received 50.4 Gy of adjuvant radiation in 28 fractions, specifically targeting the primary site of the disease. Prophylactic cranial irradiation was also implemented to mitigate the potential distant spread of the disease to the brain. Despite the administered treatment, the patient experienced disease progression around five months later and ultimately passed away 16.54 months from the time of diagnosis.

Case 6

A healthy patient was diagnosed with T3N1 small cell oesophageal carcinoma. They were treated with one cycle of carboplatin and etoposide before repeat imaging revealed disease progression merely 18 days into the initial chemotherapy cycle. The patient thereafter underwent one cycle of cyclophosphamide, doxorubicin, and vincristine (CAV) chemotherapy before it was confirmed that their disease had progressed drastically, necessitating supportive oesophageal stenting. The OS for this individual was recorded at 5.56 months from the point of diagnosis.

Case 7

A comorbid individual was diagnosed with localised T3N1 small cell oesophageal cancer. They underwent five cycles of cisplatin and etoposide and experienced chemotherapy-induced neutropaenia as a side effect. Post-treatment imaging indicated a partial response before the patient’s seemingly unexpected death 40 days later, resulting in an OS of 4.18 months from the time of diagnosis.

Case 8

A comorbid patient was diagnosed with localised T3N2 small cell oesophageal cancer and started on three cycles of carboplatin and etoposide therapy spanning six weeks. The treatment was complicated by chemotherapy-induced pancytopaenia and was sequentially followed by 50 Gy of adjuvant radiotherapy in 28 fractions, targeting the gastro-oesophageal junction (GOJ). Prophylactic cranial irradiation was additionally administered to curb the potential metastatic spread to the brain. According to available data, no post-treatment imaging was performed to assess for disease progression. The patient unfortunately passed away around six months following the completion of their radiation treatment, ultimately surviving for a total of 11.34 months from the point of diagnosis.

Case 9

A patient was diagnosed with T4N0 small cell oesophageal cancer and underwent one cycle of carboplatin therapy. However, repeat imaging revealed disease progression resulting in a PFS of five weeks. They subsequently received a short course of palliative radiotherapy, constituting 20 Gy of radiation in five fractions targeting the affected site. Moreover, the patient required supportive nasojejunal feeding to address their nutritional needs. The OS for this individual was documented at 5.16 months from the time of diagnosis.

Case 10

An individual was diagnosed with T4N1 small cell oesophageal cancer and initiated on an uncomplicated course of six cycles of carboplatin and etoposide over four months. Repeat imaging revealed a partial response to the treatment. The disease progressed three months later, resulting in a PFS of 6.08 months from the initial cycle of chemotherapy treatment. Their OS extended to 10.75 months from the point of diagnosis.

Case 11

A middle-aged patient was diagnosed with T4N1M0 small cell oesophageal cancer and initiated on neoadjuvant chemotherapy with carboplatin, which was complicated by nausea, vomiting, and generalised fatigue. Repeat imaging after two administered cycles suggested disease progression only eight weeks following the first cycle of chemotherapy. They subsequently received supportive treatment in the form of an oesophageal stent before passing away 4.18 months following the initial diagnosis.

Case 12

A relatively healthy individual was diagnosed with T4N2 small cell oesophageal cancer. Treatment with five cycles of carboplatin and etoposide over a period of three months was complicated by thrombocytopenia. CT imaging showed partial response following their first course of chemotherapy. Disease progression was evident around eight months following the start of their treatment. Despite not pursuing any second-line chemotherapy options, the patient's OS amounted to 21.86 months from their initial diagnosis.

## Discussion

This retrospective analysis of 12 cases of oesophageal carcinoma reveals a spectrum of patient demographics, comorbidities, and treatment modalities. The study additionally provides a comprehensive view of the diverse clinical trajectories and outcomes associated with the malignancy. Notably, patients with localised disease demonstrated varied responses to standard chemoradiotherapy, with some achieving remarkable OS, while others faced rapid disease progression despite aggressive intervention. The incorporation of surgery underscores the complexity of treatment decisions and the potential benefits of multidisciplinary approaches. Examining the molecular foundations of the disease, the study additionally explores the role of neuroendocrine biomarkers in guiding diagnoses and shaping the observed clinical trajectories.

Neuroendocrine malignancies, such as small cell carcinoma, are characterised by distinctive biomarkers, including chromogranin A (CgA) and synaptophysin. CgA is an intravesicular glycoprotein marker released by neuroendocrine tumours [16]. In contrast, synaptophysin is an integral membrane glycoprotein that plays a crucial role in synaptic vesicle transport and exocytosis [20]. The strategic utilisation of these biomarkers is prevalent in guiding the diagnosis of neuroendocrine malignancies. Moreover, they are crucial for assessing disease progression, gauging treatment response, and predicting overall prognosis [9,21]. The elevated expression of Ki-67, a nuclear protein associated with cellular proliferation, is a defining feature of primary small cell carcinoma [22]. The Ki-67 proliferation index, or mitotic index, typically exceeds 50% and serves as an essential diagnostic and prognostic marker in small cell disease. A high Ki-67 expression usually reflects increased tumour chemoradiosensitivity, a factor notably associated with favourable treatment outcomes and improved survival rates [10,22].

Within the examined cohort of patients, positive CgA immunostaining was observed in 55% (6/11 patients), while synaptophysin positivity was noted in 81% (13/16 patients). Significantly, all four tested patients displayed a Ki-67 index surpassing 80%, a characteristic of small cell carcinoma attributed to the high proliferation rate of the corresponding cells. Sixteen additional patients were diagnosed with advanced localised or metastatic small cell carcinoma of the oesophagus, receiving BSC due to factors like performance status and the widespread nature of the disease. The mean age of patients diagnosed with metastatic disease was 71.8 years, compared to the original cohort's average age of 67.8 years at diagnosis. Metastasis from the primary oesophageal source most frequently affected the liver, as seen in seven cases, followed by lung involvement in three cases. Interestingly, all 16 patients under BSC showed positive synaptophysin immunostaining. The predominant supportive measure employed in metastatic disease was palliative oesophageal stenting.

Among patients receiving curative treatment, seven were treated with etoposide and carboplatin, and three with etoposide and cisplatin instead. Four patients responded partially to etoposide and carboplatin, while two experienced disease progression. Alternatively, a partial response was attained in patients treated with the first-line cisplatin and etoposide regimen. Two patients with advanced disease, treated exclusively with carboplatin, displayed inferior OS and PFS in contrast to those treated with etoposide and carboplatin, showing considerably better outcomes. For metastatic disease, four patients received etoposide and carboplatin as per the guidelines set by the European Neuroendocrine Tumour Society.

Five patients underwent treatment with second-line chemotherapy, primarily with cyclophosphamide, doxorubicin and vincristine (CAV). Unfortunately, all four patients on second-line CAV chemotherapy experienced disease progression. One patient was treated with second-line etoposide and carboplatin after a nine-month platinum-free interval, achieving a partial response. This prompted the administration of third-line treatment with carboplatin upon further disease progression. Three patients received sequential radical radiotherapy, around one to three months after completing their recommended course of neoadjuvant chemotherapy. While adjuvant radiation therapy demonstrated favourable outcomes in certain instances by consolidating the initial response achieved through chemotherapy and impeding further progression, the sustained impact varied among these patients, underscoring the intricate nature of treatment responses in SCEC. None of the patients receiving PCI developed brain metastasis from the primary source of the disease.

The median age of patients in this study was 69 years. For patients with non-metastatic disease undergoing treatment, the median OS was 12.01 months. The choice of utilising the median over the mean in conveying the OS data among treated patients with non-metastatic disease stems from the notable survival of three patients at the conclusion of this study. A major limitation of this study lies in the lack of follow-up of the surviving patients beyond 2020, which may impact the precision of the OS data. In contrast, patients with treated metastatic disease exhibited a mean OS of 9.15 months, whereas those managed with BSC demonstrated an average survival of 2.55 months. These findings underline the intricate landscape of the condition and highlight the need for further research and optimisation in the management of advanced localised disease.

## Conclusions

SCEC is a rare, aggressive neuroendocrine malignancy that exhibits distinctive histological traits and carries a guarded prognosis. This study's findings underscore the complexity of therapeutic decisions and indicate that a combination of chemoradiotherapy and radical surgery holds promise in achieving local control in non-metastatic disease. The ongoing discourse regarding the introduction of surgery and radiotherapy for localised disease highlights the need for a more comprehensive and tailored approach to treatment. A notable issue in the current landscape of the condition is the absence of clear guidelines, especially concerning combined treatment modalities such as chemoradiotherapy and surgery. While the European Neuroendocrine Tumour Society provides some direction, the dearth of clinical trials studying diverse treatments for SCEC renders the evidence base weak. This emphasises the urgent need for further research to delineate optimal treatment approaches and establish comprehensive guidelines for this challenging malignancy.
